# Discrimination of Two Cultivars of *Alpinia Officinarum* Hance Using an Electronic Nose and Gas Chromatography-Mass Spectrometry Coupled with Chemometrics

**DOI:** 10.3390/s19030572

**Published:** 2019-01-30

**Authors:** Qin Long, Zhong Li, Bin Han, Hamid Gholam Hosseini, Huaying Zhou, Shumei Wang, Dehan Luo

**Affiliations:** 1 College of Traditional Chinese Medicine, Guangdong Pharmaceutical University, Guangzhou 510006, China; qinlong28@163.com (Q.L.); hblz99@21cn.com (B.H.); gdpuwsm@126.com (S.W.); 2 School of Engineering, Computer and Mathematical Sciences, Auckland University of Technology, Private Bag 92006, Auckland 1142, New Zealand; hamid.gholamhosseini@aut.ac.nz; 3 College of Medical Information Engineering, Guangdong Pharmaceutical University, Guangzhou 510006, China; zhhying1224@163.com; 4 Key Laboratory of Digital Quality Evaluation of Chinese Materia Medica, State Administration of Traditional Chinese Medicine, Guangzhou 510006, China; 5 School of Information Engineering, Guangdong University of Technology, Guangzhou 510006, China; dehanluo@gdut.edu.cn

**Keywords:** *A. officinarum*, Zhutou galangal, Fengwo galangal, sensors, E-nose, GC-MS, PCA, PLS

## Abstract

*Background*: *Alpinia officinarum* Hance is both an herbal medicine and a condiment, and generally has different cultivars such as Zhutou galangal and Fengwo galangal. The appearance of these *A. officinarum* cultivars is similar, but their chemical composition and quality are different. It is therefore important to discriminate between different *A. officinarum* plants to ensure the consistency of the efficacy of the medicine. Therefore, we used an electronic nose (E-nose) to explore the differences in odor information between the two cultivars for fast and robust discrimination. *Methods*: Odor and volatile components of all *A. officinarum* samples were detected by the E-nose and gas chromatography-mass spectrometry (GC-MS), respectively. The E-nose sensors and GC-MS data were analyzed respectively by principal component analysis (PCA), the correlation between E-nose sensors and GC-MS data were analyzed by partial least squares (PLS). *Results*: It was found that Zhutou galangal and Fengwo galangal can be discriminated by combining the E-nose with PCA, and the E-nose sensors S2, S6, S7, S9 were important sensors for distinguishing different cultivars of *A. officinarum*. A total of 56 volatile components of *A. officinarum* were identified by the GC-MS analysis, and the composition and content of the volatile components from the two different *A. officinarum* cultivars were different, in particular the relative contents of 1,8-cineole and α-farnesene. The classification result by PCA analysis based on GC-MS data was consistent with the E-nose results. The PLS analysis demonstrated that the volatile terpene, alcohol and ester components primarily interacted with the sensors S2 and S7, indicating that particular E-nose sensors were highly correlated with some aroma constituents. *Conclusions*: Combined with advanced chemometrics, the E-nose detection technology can discriminate two cultivars of *A. officinarum*, with GC-MS providing support to determine the material basis of the E-nose sensors’ response.

## 1. Introduction

*Alpinia officinarum* Hance (galangal) belongs to the Zingiberaceae family, and is widely grown in Southeast China (Guangdong, Guangxi, Hainan and Yunnan provinces), India, and Southeast Asian countries such as Thailand, Indonesia, and Philippines. The rhizomes of this plant can be used as a condiment for foods, and a traditional medicine for several purposes, such as a stomachic in China, or for carminative, anti-flatulent, antifungal, and anti-itching applications in Thailand [1]. According to recent pharmacological research reports, the rhizomes of *A. officinarum* indeed have various pharmacological benefits such as antimicrobial, antiviral, antitumour, anti-oxidant, anti-inflammatory, anti-ulcer, and anticoagulation activities [2].

In China, the Daodi origin of *A. officinarum* is Xuwen Guangdong, where most of *A. officinarum* is artificially cultivated. Generally, it has different cultivars. Zhutou galangal and Fengwo galangal are two of these cultivars, and it is believed that the quality of Fengwo galangal is better [3]. Our previous study found differences in the chemical composition of Zhutou galangal and Fengwo galangal. Particularly, the average contents of galangin in Zhutou galangal and Fengwo galangal are 0.30%, 0.98%, respectively [4]. Galangin is one of the most important and naturally active flavonoids in *A. officinarum* [5]. Many studies have reported on the pharmacological effects of galangin, such as anti-cancer [5,6], anti-inflammatory [7,8], anti-bacterial [9], anti-oxidation properties [10], etc. Therefore, it is suggested that the clinical efficacy of Fengwo galangal is better than that of Zhutou galangal, because the content of galangin in Fengwo galangal is higher. Consequently, the identification of the two *A. officinarum* cultivars is important in ensuring that high-quality *A. officinarum* be selected for medicinal use. However, the appearance traits of the two *A. officinarum* cultivars are very similar, and it is difficult for inexperienced people to discriminate between the two. Typical chemical composition analysis method, such as high-performance liquid chromatography (HPLC) and gas chromatography-mass spectrometry (GC-MS), may identify different *A. officinarum* cultivars, but the pre-treatment process is cumbersome, time consuming and costly. Therefore, there is a need to find fast, efficient and non-destructive methods to distinguish between the different cultivars of *A. officinarum*.

The electronic nose (E-nose) is a new type of analytical testing equipment that simulates the human olfactory system. It is composed of three parts: (1) a sample handling system; (2) a detection system that is made up of an array of gas sensors with partial specificity; (3) an odor data processing system. E-noses can rapidly detect the overall odor characteristics of samples, transform them into digital signals and directly analyze them using the provided software, so that we can intuitively and rapidly assess the results. Compared to traditional odor analysis methods, E-noses are easily built systems that offer simple sample pre-treatment, non-destructive features, relatively fast assessment detection, a wide odor operating range, and generally high sensitivity and selectivity to the tested odorants [11]. Nowadays, E-nose technology have been successfully applied in different fields, such as quality assessment of food products [12,13], medical diagnostics [14,15], as well as in environmental monitoring [16,17]. Because traditional Chinese medicines generally have a particular smell, and the odor can reflect the quality of the medicinal material, the E-nose technology has gradually been adopted to assess the quality of Chinese medicines, for example, it can be applied to the discrimination of origin [18], authenticity [19], harvesting time [20], and quality grades [21]. *A. officinarum* as an aromatic medicinal material, and the overall odor information of Zhutou galangal and Fengwo galangal may be different (they are difficult to distinguish by the human sense of smell), so we believe that the detection of *A. officinarum*’s odors by an E-nose could be a feasible way to identify them.

Therefore, this study employed an E-nose to detect the odors of two different cultivars of *A. officinarum*—Zhutou galangal and Fengwo galangal—and then the odor information was processed by multivariate statistical analysis methods to realize the rapid identification of the different cultivars of *A. officinarum*. In addition, to further explore the material basis responsible for the odor information differences between the two cultivars *A. officinarum*, we used GC-MS to study the volatile oil components of Zhutou galangal and Fengwo galangal, then the correlation between the volatile components and E-nose odor information was studied to explore what volatile components might affect the E-nose sensors’ responses.

## 2. Materials and Methods

### 2.1. A. officinarum Material

All *A. officinarum* samples were collected from different farmers of Xuwen Guangdong. Five of the samples were Zhutou galangal (1ZT, 2ZT, 3ZT, 4ZT, 5ZT); and ten samples were Fengwo galangal (1FW, 2FW, 3FW, 4FW, 5FW, 6FW, 7FW, 8FW, 9FW, 10FW). All of these samples were authenticated by Associate Professor Zhong Li (College of Traditional Chinese Medicine, Guangdong Pharmaceutical University, Guangzhou, China).

### 2.2. E-Nose Equipment and Measurements

The E-nose instrument used in this experiment is the PEN3, a portable E-nose made by the German company AIRSENSE (Schwerin, German). The PEN3 E-nose is an analytical instrument that consists of a set of complex chemical sensors and recognition software. It has 10 metal oxide semiconductor (MOS) sensors, namely S1, S2, S3, S4, S5, S6, S7, S8, S9 and S10. Each sensor has different detection sensitivity, as shown in Table 1. The sensor response is defined as the ratio of conductance: G/G_0_ (where G represents the resistance of each sensor in the chamber after exposure to a target gas and G_0_ represents the resistance when each sensor is exposed to zero gas filtered by standard activated carbon) [20]. The software provided is mainly composed of Winmaster, an E-nose package developed by AIRSENSE. The software realizes data collection and other automatic control functions during the E-nose working process, and also can analyze and process the collected data.

E-nose measurements: The samples were accurately weighed to 15 g and then placed in 150 mL headspace vials. The headspace generation time is 30 min. Then sample gas was injected into the testing chamber through a syringe at a flow rate of 150 mL/min. The sampling time was set to 120 s and the sampling interval was set to 1 s, the cleaning time of the sensor array was set to 120 s. The response value of each sensors for sample was recorded, and response curves were generated. Every sample was continuously sampled four times, a total of 60 sample sets (15 × 4) were obtained. Before the sample detection, the repeatability of the test method should be evaluated. Based on the method mentioned above, we performed six parallel tests on sample of 1FW, and calculated the relative standard deviation (RSD, *n* = 6) of the maximum response value for each sensor.

### 2.3. GC-MS Analysis

#### 2.3.1. Preparation of Volatile Oil 

The dried rhizomes of *A. officinarum* were crushed into powder and passed through a 20 mesh sieve; 100 g of the powder was weighed and 1000 mL water was added, then the volatile oils of *A. officinarum* were extracted for 5 h by the steam distillation method. The obtained volatile oil was dried over anhydrous sodium sulfate and allowed to stand overnight. The volatile oil was diluted 50 times with ethyl acetate and used in further GC-MS determination.

#### 2.3.2. The GC-MS Parameters and Conditions 

The samples of *A. officinarum* volatile oil were analyzed on an Agilent 7890B gas chromatography system coupled to an Agilent 5977A MSD system (Agilent Technologies Inc., Santa Clara, CA, USA). GC conditions: Capillary column HP-5MS (30 m × 0.25 mm × 0.25 µm) was utilized to separate the volatile oil components. Helium was used as the carrier gas at a constant flow rate of 1 mL/min through the column. The injector temperature was maintained at 250 °C. Injection volume was 1 µL by split mode (split ratio is 50:1). The oven temperature was programmed as follows: The initial oven temperature was 70 °C, held for 3 min, ramped to 100 °C at a rate of 3 °C/min, held for 3 min, and then ramped to 120 °C at 10 °C/min, to 140 °C at a rate of 2 °C/min, held for 3 min, finally ramped to 220 °C at 10 °C/min. MS conditions: EI source, electron energy of 70 eV, ionization temperature 230 °C, interface temperature at 280 °C, The temperature of MS quadrupole 150 °C, quantity scanning range was from 20 amu to 500 amu.

### 2.4. Statistical Processing

For the E-nose, we randomly selected 45 samples (Zhutou galangal: 5 × 3; Fengwo galangal: 10 × 3) from the total sample collection as the training set, and the remaining 15 samples (Zhutou Galangal: 5 × 1; Fengwo Galangal: 10 × 1) were used as the testing set. The E-nose data of each sample was too large, the extraction of feature values must be performed first. Feature values include the following: response values for each sensor at certain time points, mean value of each sensor response, the maximum value of each sensor response, the variance of each sensor’s response value, etc. Through continuous experimentation, we finally decided to extract the response values of each sensor at 15th second and maximum response value of each sensor as feature values for principal component analysis (PCA). PCA is a well-known technique used for reducing the dimensionality of data, calculating a number of variables that best describe the differences between the samples and allow visualizing of cluster, so according to the score plot and factor loading plot obtained from PCA, we can achieve the classification of two cultivars *A. officinarum* and find what sensors are playing an important role in distinguishing two *A. officinarum.* All the above data pre-processing and analysis were performed using MATLAB R2016a (MathWorks, Natick, MA, USA).

For GC-MS, the compounds were identified by NIST (NIST 14. L, National Institute of Standards and Technology, Gaithersburg, MD, USA) library search data system (matching degree was over 85%) and reference related literature. The quantification of each compound is carried out by peak area normalization, the relative content of compounds of one cultivar *A. officinarum* was recorded as mean ± standard, and the relative content of the each component was tested by *t*-test to check whether the relative content had significant difference between the two *A. officinarum* cultivars. Then, some of the volatile components were selected for PCA analysis to distinguish the two *A. officinarum* cultivars and to find important compounds that distinguish between the two. The criteria for selecting volatile components were as follows: (1) for common compounds of two kinds of *A. officinarum*, the relative content of each component was analyzed by *t*-test, and then the compounds with significant differences selected; (2) all the specific compounds were chosen, because they were only detected in one *A. officinarum* and were considered important to differentiating between these two. 

For the correlation between E-nose and GC-MS data, partial least squares (PLS) is generally used for regression analysis of multi-dependent variables and multi-independent variables, and also helps us to judge the correlation between independent variables and dependent variables [22]. Therefore, taking the relative content of the selected volatile components as an independent variable and the maximum response value of 10 sensor as the dependent variable, PLS was applied to correlate the volatile components of *A. officinarum* with the E-nose odor information to find some chemical components that might affect the response of the E-nose sensors. The proposed PCA and PLS analysis were performed by Unscrambler Software, version 10.4 (Camo Analytics, Oslo, Norway).

## 3. Results

### 3.1. Application of the E-Nose to the Odor of Two A. officinarum Cultivars 

#### 3.1.1. Repeatability of E-Nose Experiment

As shown in Table 2, the RSD values of all sensors response to 1FW were less than 5%, indicating that the experiment has good repeatability, that is, the sensors did not exhibit a “memory” to prior exposure sample, cleaning in fresh air always brought the sensors back to approximately same resistance as initial. Therefore, it was found that the use of E-nose in detecting *A. officinarum* odor resulted in good repeatability, sensitivity, effectiveness and stability.

#### 3.1.2. E-nose Response of the Two *A. officinarum* Cultivars 

**Figure 1:**
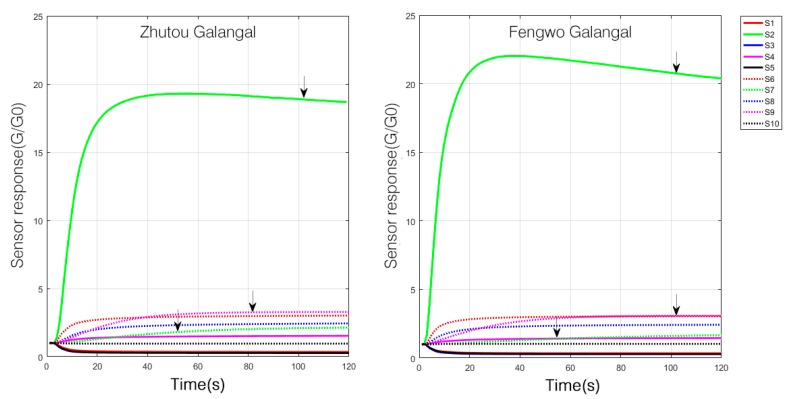
Sensor response of Zhutou galangal and Fengwo galangal.

The sensor responses of both Zhutou galangal and Fengwo galangal are shown in Figure 1. It appeared that the sensors’ responses to the two different cultivars were similar, which indicated that the odors of the two cultivars of *A. officinarum* are similar, but there were still differences in the response curves for some sensors. For example, the response value of S2 to Fengwo galangal was significantly higher than that of Zhutou Galangal, while the response value of S7 to Fengwo galangal was lower than that to Zhutou galangal, and the response of S9 was higher than that of S6 for Zhutou galangal, but an almost equal response was observed for Fengwo galangal. 

**Figure 2:**
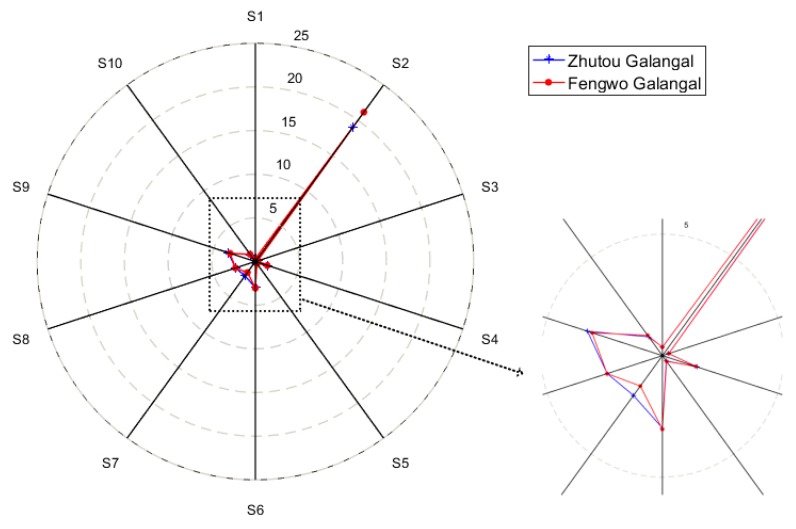
Radar plots of Zhutou galangal and Fengwo galangal.

In the radar plots, as shown in Figure 2, the difference of the responses of sensors S2, S7, and S9 between the two cultivars were confirmed again. In general, the Zhutou galangal and Fengwo galangal cannot be effectively and quickly distinguished by the response curves and the radar charts. Therefore, it is necessary to use multivariate statistical analysis to process the E-nose sensors’ response data, mining more effective information to accurately identify different *A. officinarum* cultivars.

#### 3.1.3. Discrimination between the Two *A. officinarum* Cultivars by PCA

**Figure 3:**
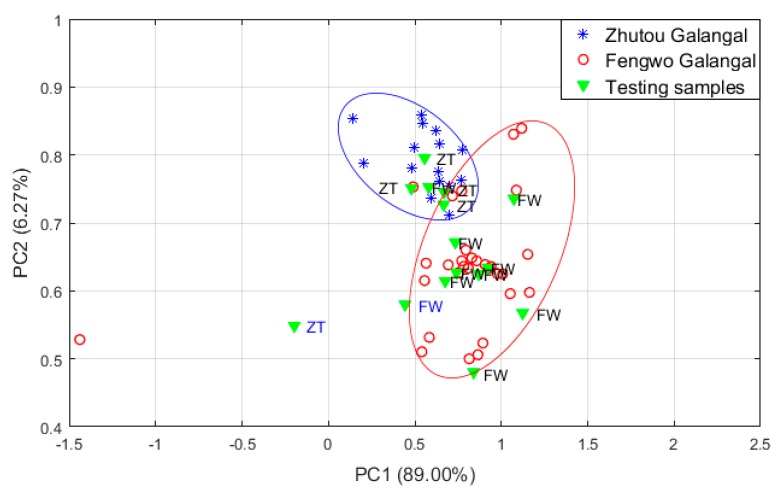
PCA classification results based on E-nose data of two cultivars *A. officinarum*. (ZT, FW are the labeling of the identification results of test samples, ZT, FW respectively represent Zhutou galangal and Fengwo galangal, and the result of blue font indicates misidentification).

The PCA was conducted as shown in Figure 3. Most samples of Zhutou galangal and Fengwo galangal are well separated, and the first two PCs accounted for 95.27% of the total variance, indicating that the PCA analysis retained most of the information in the original data. The remaining 15 test samples were introduced into the PCA algorithm as unknown samples to verify the correct recognition rate which is defined as the ratio of the number of test samples correctly identified and the number of total test samples. We used the Euclidean distance analysis method to calculate the distance between unknown samples and training samples of different classes, and to classify the unknown samples into the class with the smallest distance, thereby the properties of unknown samples were predicted. The results in Figure 3 indicate that one Zhutou galangal sample and one Fengwo galangal sample were misidentified, so the recognition rate of PCA algorithm for different cultivars *A. officinarum* is 86%.

**Figure 4:**
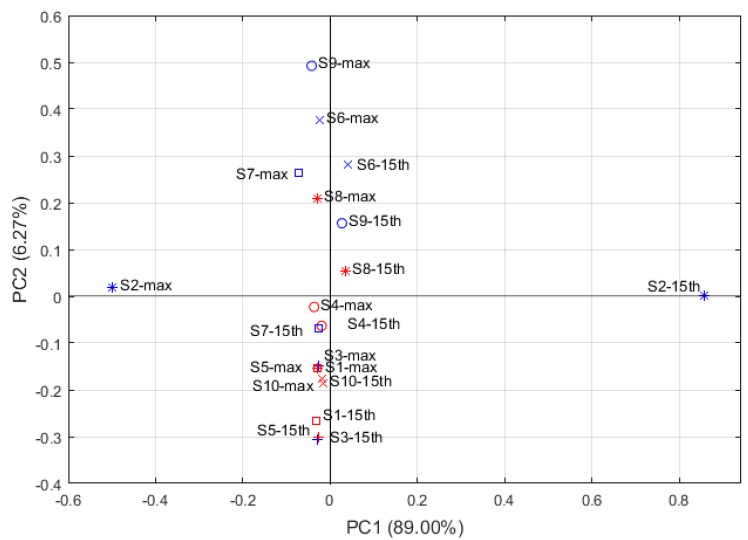
Loading plot of sensors (S1-15th~S10-15th represent the factors of response value of the sensors S1~S10 at the 15th second respectively; S1-max~S10-max represent the factors of maximum response value of the sensors S1~S10 respectively).

Figure 4 is a factor loading plot obtained from PCA, which shows the relationship between the variables and how much they influenced the system. Therefore, the loading analysis might help to identify the important factors for the discrimination of different clusters *A. officinarum*. Sensors with loading parameters near to zero for a particular principal component have a low contribution to the total response of the array, whereas high values indicates a discriminating sensor. Figure 4 shows that the load parameters of S2-15th and S2-max are higher in the PC1, while the load parameters of S9-max, S6-max, S6-15th and S7-max are higher in the PC2. Therefore, S2, S6, S7, S9 are important sensors for distinguishing different cultivars of *A. officinarum*. These results are consistent with the analysis of the odor response curve and the odor radar chart data discussed in Section 3.1.2.

### 3.2. Investigation of the GC-MS Data from Two A. officinarum Cultivars 

#### 3.2.1. Identification and Comparison of Volatile Compounds between Zhutou Galangal and Fengwo Galangal

According to the above E-nose odor analysis, there are differences in the odors of different cultivars of *A. officinarum*. However, these odor differences must be caused by differences in their inherent volatile components, so it is necessary to analyze the volatile components of different *A. officinarum* cultivars by GC-MS to find what main components can cause these odor differences.

**Figure 5:**
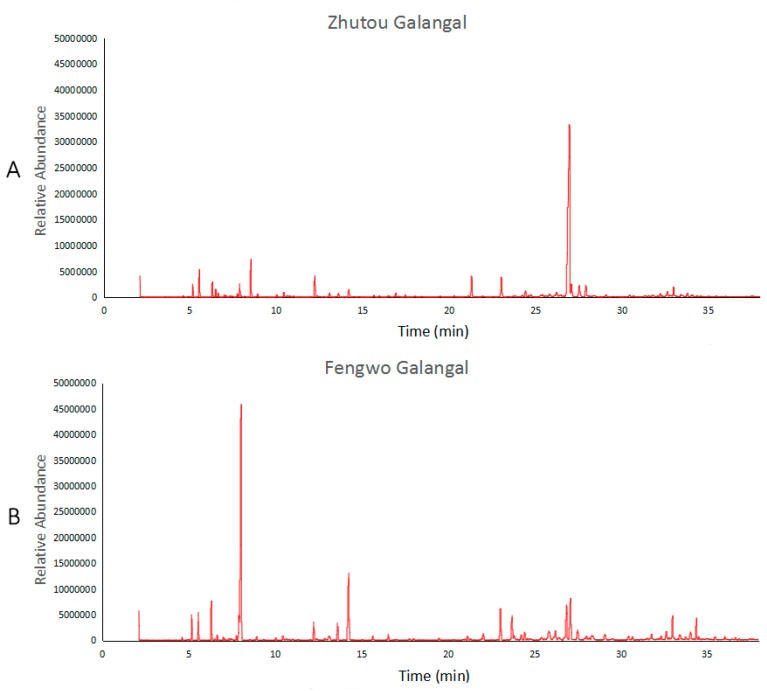
Total ion current chromatogram of volatile oil from Zhutou galangal and Fengwo galangal. (**A**) is the total ion current chromatogram from sample 1ZT, (**B**) is the total ion current chromatogram from sample 2FW).

The total ion current chromatogram of the volatile oils of Zhutou galangal and Fengwo galangal are shown in Figure 5. The composition and content of the volatile oil components between the two *A. officinarum* cultivars are different, the retention time of the component with the highest content in Zhutou galangal is 26.981 min, while for Fengwo galangal it is 8.031 min. Table 3 shows the composition details of the volatile oils of Zhutou galangal and Fengwo galangal by GC-MS which can be used for the identification and comparison of the volatile oil components of the two *A. officinarum* cultivars. 

Table 3 shows that a total of 56 compounds were identified in the two *A. officinarum* cultivars, and there were 52 and 43 compounds in Zhutou galangal and Fengwo galangal respectively, which were mainly composed of terpenes, alcohols, esters and others. For terpenes, both Zhutou galangal and Fengwo galangal are rich in terpenes, and the relative content in Zhutou galangal was 76.44%, and in Fengwo galangal it was 74.53%. A total of 32 terpene compounds were identified in the two *A. officinarum* cultivar samples, of which 24 were common compounds, five were specific to Zhutou galangal, and three were specific to Fengwo galangal. Among the 24 common terpene compounds, the relative contents of 12 components between the two *A. officinarum* cultivars were significantly different. The compounds with the most different relative contents were α-farnesene (highest in Zhutou galangal) and 1,8-cineole (highest in Fengwo galangal), α-Farnesene in Zhutou galangal (42.65%) was almost seven times higher than that in Fengwo galangal (6.00%); while 1,8-cineole in Fengwo galangal (29.13%) was almost 79 times higher than that in Zhutou galangal (0.37%). It is worth noting that α-farnesene is one of the aroma components for many fruits (such as apples, bananas, and pears) [23,24], while the smell of 1,8-cineole is defined as cool and similar to camphor, so the two compounds may be important substances that cause the difference in the odors of two kinds of *A. officinarum*. Other common terpene compounds with obvious different contents were D-limonene, camphor, α-*trans*-bergamotene, (±)-γ-cadinene and so on. The special terpenes in Zhutou galangal are β-ocimene, α-cubebene, alloaromadendrene, etc., of which the one with the higher content was α-cubebene (3.43%); the special terpenes in Fengwo galangal were epizonarene, γ-selinene, and selina-3,7(11)-diene, with the higher content corresponding to selina-3,7(11)-diene (1.18%). Therefore, it is concluded that there are great differences in the composition and content of terpenoids between the two *A. officinarum* cultivars, which may be important compounds that cause the odor differences between Zhutou galangal and Fengwo galangal.

For alcohol compounds, the relative content in Zhutou galangal was 12.45%, and in Fengwo galangal it was 19.82%. A total of 14 alcohol compounds were identified in the two *A. officinarum* cultivar samples, of which 11 were common alcohol compounds and three were specific to Zhutou Galangal. Among the 11 common alcohol compounds, the relative content of eight components were significantly different between the two *A. officinarum* cultivars. The compound with the most different relative content was α-terpineol, which in Fengwo Galangal (9.54%) was almost seven times higher than that in Zhutou Galangal (1.45%). Terpineol has a lilac aroma, and the different content of terpineol in the two kinds of *A. officinarum* may be important for the odor difference between Zhutou galangal and Fengwo galangal.

For ester compounds, the relative content in Zhutou galangal was 1.13%, and in Fengwo Galangal it was 1.50%. A total of five compounds were identified, among which isobutyl 2-methylbutyrate and fenchyl acetate were common ester compounds, whereas 2-methylbutyl-2-methylbutyrate and 2-methylbutyl-3-methylbutanoate were specific to Zhutou galangal, and phenethyl butyrate was specific to Fengwo galangal. 

Among other aromatic, ketone, and aldehyde components, the ketone methyl-5-hepten-2-one was specific to Zhutou galangal, with a relative content of 1.25%, and this may be an important component to distinguish between the two *A. officinarum* cultivars The relative content of other compounds were lower, so these compounds should have less effect to the odor differences of the two cultivars.

#### 3.2.2. Analysis of Volatile Compounds of Two *A. officinarum* Cultivars by PCA

**Figure 6:**
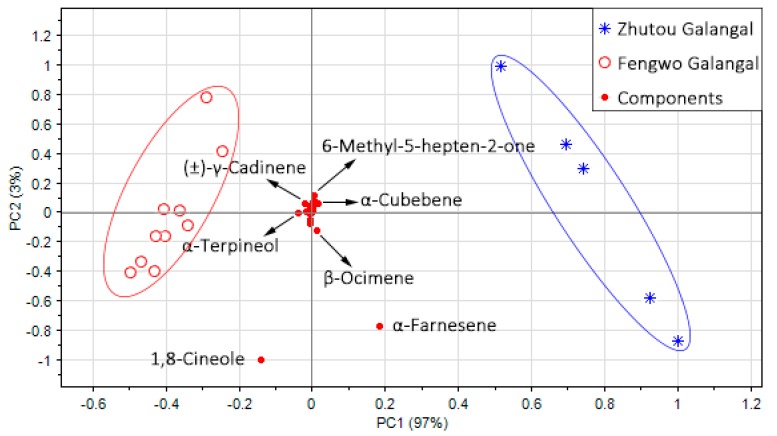
Bioplot of PCA based on GC-MS data of the two *A. officinarum* cultivars.

According to the two selection criteria described in Section 2.4., a total of 38 possible aroma compounds were selected (see Table 3), of which 20 were terpenes, 11 were alcohols, three were esters, and four belonged to other classes. PCA was performed based on all these components, and the corresponding bioplot is shown in Figure 6. It can be seen that the Zhutou galangal and Fengwo galangal samples were scattered significantly, with the first two PCs (the variance contribution rate was 100%), and the projections of 1,8-cineole and α-farnesene on the first principal component were significantly higher than those of the other components, so 1,8-cineole, and α-farnesene were the most important components to distinguish the two kinds of *A. officinarum*. The compounds α-terpineol, (±)-γ-cadinene, β-ocimene, α-cubebene, and 6-methyl-5-hepten-2-one also played an important role in distinguishing the two kinds of *A. officinarum*, while other components were almost focused on the origin, so their contribution to distinguishing between the two kinds of *A. officinarum* was not obvious. It was proved again that the terpene components are the main substance for distinguishing Zhutou galangal and Fengwo galangal, and these substances may cause the differences in the odors of the two *A. officinarum* cultivars, so the next step was to study the relationship between the volatile components and the E-nose odor responses to explore the effect of these components on the sensors’ response.

### 3.3. Correlation between E-Nose and GC-MS Data

**Figure 7:**
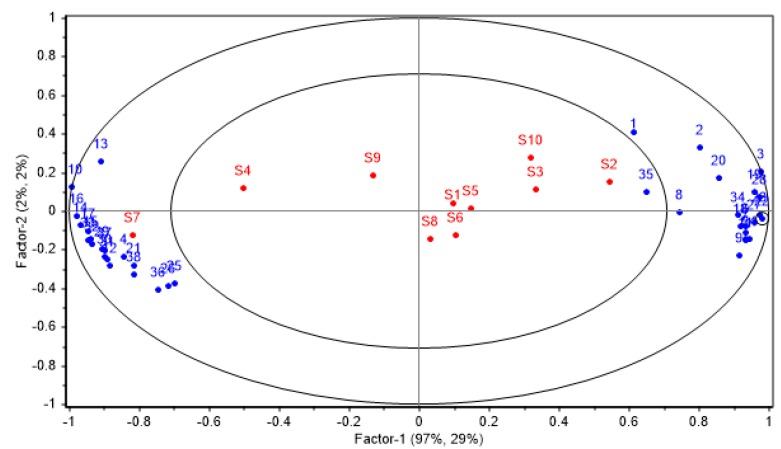
Bioplot for the correlation between volatile components and the E-nose sensor responses (1~38: the 38 selected compounds, the numbers are same as the selected compounds in Table 3).

In order to explore the material basis of the *A. officinarum* E-nose responses, it is necessary to investigate the correlation between the E-nose and GC-MS results. The PLS was performed with the 38 selected compounds as independent variables and the maximum response values of 10 sensors as dependent variables, and the corresponding bioplot of the PLS is presented in Figure 7. The shorter the distance between a compound and the characteristic value of a sensor, the higher the correlation of this compound with this sensor response. As shown in the Figure 7, all compounds are divided into two groups, which were respectively distributed on the left and right sides of the coordinate system. The compound group on the right was closest to S2, indicating that the sensor S2 response has a high positive correlation with these compounds, that is, the change of sensor S2 response value is greatly affected by these compounds, which include (-)-α-pinene (1), D-limonene (2), isoledene (8), benzylacetone (35) and so on. The left compound group was closest to S7, indicating that the sensor S7 response has a high positive correlation with these compounds, which include as D-limonene (2), camphor (4), (+)-δ-cadinene (12),linalool (21) and so on. This result was highly associated with the PCA result based on E-nose data, because it was also proved that S2 and S7 were important sensors for identifying different *A. officinarum* cultivars. Therefore, it may be concluded that these terpenes, alcohols, and esters mainly interact with the sensors S2 and S7, thereby producing different odor response values for the two *A. officinarum* cultivars. However, in this bioplot, the response of other sensors affected by what components are not well shown, which needs to be further explored in future research. 

## 4. Conclusions

In this study, the aroma characteristics of Zhutou galangal and Fengwo galangal were investigated by comprehensive analysis of odor and volatile components using an E-nose and GC-MS. The E-nose showed a good performance in classifying the two *A. officinarum* cultivars based on PCA analysis, which indicates that there are differences in odor characteristics between Zhutou galangal and Fengwo galangal. Two E-nose sensors, S2 and S7, were important sensors to distinguish the two *A. officinarum* cultivars. After further study of the volatile components by GC-MS, a total of 56 compounds were identified in the two *A. officinarum* cultivars, but the composition and content of volatile components in Zhutou galangal and Fengwo galangal were different, in particular the relative contents of 1,8-cineole and α-farnesene were the most obvious differences. Therefore, the differences between Zhutou galangal and Fengwo galangal odor were possibly caused by the volatile components. To explore the effects of volatile components on the response of the E-nose sensors, PLS was applied to correlate the odor information of *A. officinarum* with the volatile components. It was found that the volatile terpene, alcohol, and ester components mainly interact with sensors S2 and S7, this implies that these volatile components might induce the response of sensors S2 and S7 to change considerably and thus enable the E-nose to differentiate between Zhutou galangal and Fengwo galangal successfully.

In traditional Chinese medicine, the quality and efficacy of most Chinese medicines will change depending on the cultivar [25], as illustrated by the examples of licorice [26], rehmannia [27], Citri Reticulatae Pericarpium [28], Mu Dan Pi [29], etc. The results of this study demonstrated that the E-nose technology combined with chemometrics can realize the identification of two *A. officinarum* cultivars, and suggests that future research work could combine E-nose data with chemometrics to identify other confusing Chinese medicines or plants, and provide rapid, easy-to-operate, accurate and non-destructive identification methods to ensure the efficacy of traditional Chinese medicine. This study also employed GC-MS technology to explore the material basis of the E-nose responses to *A. officinarum* odors, which provided an experimental basis for the study of the odor-causing material basis of this Chinese medicine and its reaction mechanism with E-nose sensors.

## Figures and Tables

**Table 1:** The components and main applications of the sensors of the PEN3.

No.	Detection of Chemical Components
S1	Aromatic
S2	Nitrogen Oxides
S3	Ammonia, aromatic
S4	hydrogen
S5	Alkanes, aromatic ingredients
S6	Methane
S7	Sulfide
S8	Ethanol
S9	Aromatic ingredients, organic sulfur compounds
S10	Alkanes

**Table 2:** The Repeatability of response values of E-nose 10 sensors (*n* = 6).

Sensor	S1	S2	S3	S4	S5	S6	S7	S8	S9	S10
RSD/%	0.93	3.40	0.80	0.65	0.79	4.20	3.15	3.98	4.13	0.74

**Table 3:** Identified volatile compounds of Zhutou galangal and Fengwo galangal by GC-MS.

Peak No.	Category	Compound	CAS	Relative Content (%), Mean ± Standard		Selected ^1^
				Zhutou Galangal	Fengwo Galangal	
1	Terpenes	Camphene	79-92-5	1.83 ± 0.86	1.79 ± 0.67	
2		β-Pinene	127-91-3	1.36 ± 0.56	2.23 ± 0.86	
3		β-Myrcene	123-35-3	0.326 ± 0.09	0.34 ± 0.11	
4		γ-Terpinene	99-85-4	0.28 ± 0.11	0.32 ± 0.04	
5		Terpinolene	586-62-9	0.23 ± 0.05	0.29 ± 0.08	
6		β-Caryophyllene	87-44-5	3.48 ± 0.038	3.55 ± 1.14	
7		α-Caryophyllene	6753-98-6	1.13 ± 0.08	1.02 ± 0.20	
8		γ-Muurolene	30021-74-0	0.60 ± 0.11	0.79 ± 0.31	
9		α-Selinine	473-13-2	1.87 ± 0.25	2.06 ± 0.26	
10		Germacrene B	15423-57-1	0.50 ± 0.15	1.09 ± 0.67	
11		Calarene	17334-55-3	0.41 ± 0.10	0.51 ± 0.04	
12		α-Elemene	5951-67-7	0.42 ± 0.13	0.39 ± 0.03	
13		(-)-α-Pinene	7785-26-4	0.78 ± 0.40	1.50 ± 0.65 *	1
14		d-Limonene	5989-27-5	1.27 ± 0.29	2.42 ± 0.59 *	2
15		1,8-Cineole	470-82-6	0.37 ± 0.44	29.13 ± 4.16 *	3
16		Camphor	464-49-3	2.87 ± 0.41	1.78 ± 0.10 *	4
17		Ylangene	14912-44-8	0.24 ± 0.04	0.52 ± 0.05 *	5
18		α-*trans*-Bergamotene	13474-59-4	0.27 ± 0.05	3.40 ± 0.43 *	6
19		α-Guaiene	3691-12-1	0.29 ± 0.04	0.79 ± 0.09 *	7
20		Isoledene	95910-36-4	0.46 ± 0.04	0.77 ± 0.15 *	8
21		β-Selinene	17066-67-0	1.16 ± 0.22	2.33 ± 0.25 *	9
22		α-Farnesene	502-61-4	42.65 ± 9.83	6.00 ± 1.47 *	10
23		( ± )-γ-Cadinene	39029-41-9	2.98 ± 0.46	7.15 ± 0.71 *	11
24		(+)-δ-Cadinene	483-76-1	3.22 ± 0.51	1.75 ± 0.18 *	12
25		β-Ocimene	13877-91-3	2.44 ± 1.35	nd	13
26		α-Cubebene	17699-14-8	3.43 ± 0.42	nd	14
27		Alloaromadendrene	25246-27-9	0.52 ± 0.09	nd	15
28		Cadina-1(6),4-diene	16729-00-3	0.31 ± 0.04	nd	16
29		1ξ,6ξ,7ξ-Cadina-4,9-diene	31983-22-9	0.73 ± 0.15	nd	17
30		Epizonarene	41702-63-0	nd	0.71 ± 0.14	18
31		γ-Selinene	515-17-3	nd	0.72 ± 0.11	19
32		Selina-3,7(11)-diene	6813-21-4	nd	1.18 ± 0.43	20
33	Alcohols	Borneol	507-70-0	0.68 ± 0.18	0.69 ± 0.35	
34		α-Cadinol	481-34-5	2.30 ± 0.74	1.86 ± 0.55	
35		β-Bisabolol	15352-77-9	0.67 ± 0.23	0.74 ± 0.13	
36		Linalool	78-70-6	0.73 ± 0.13	0.45 ± 0.03 *	21
37		Terpinen-4-ol	562-74-3	0.62 ± 0.11	1.89 ± 0.8 *	22
38		α-Terpineol	98-55-5	1.45 ± 0.55	9.54 ± 0.82 *	23
39		Epicubenol	19912-67-5	0.45 ± 0.09	0.75 ± 0.05 *	24
40		T-Cadinol	5937-11-1	1.60 ± 0.38	0.86 ± 0.28 *	25
41		α-Bisabolol	515-69-5	0.90 ± 0.31	0.38 ± 0.07 *	26
42		Juniper camphor	473-04-1	0.39 ± 0.19	1.18 ± 0.14 *	27
43		α-trans-Bergamotenol	88034-74-6	0.32 ± 0.13	1.48 ± 0.18 *	28
44		Geraniol	106-24-1	0.44 ± 0.11	nd	29
45		Copaborneol	21966-93-8	0.45 ± 0.12	nd	30
46		Epicubenol	19912-67-5	0.73 ± 0.20	nd	31
47	Esters	Isobutyl 2-methylbutyrate	2445-67-2	0.19 ± 0.05	0.22 ± 0.04	
48		Fenchyl acetate	13851-11-1	0.46 ± 0.29	0.48 ± 0.13	
49		2-Methylbutyl-2-methyl-butyrate	2445-78-5	0.30 ± 0.05	nd	32
50		2-Methylbutyl-3-methyl-butanoate	2445-77-4	0.18 ± 0.02	nd	33
51		Phenethyl butyrate	103-52-6	nd	0.80 ± 0.19	34
52	Others	*o-*Cymene	527-84-4	0.46 ± 0.15	0.47 ± 0.10	
53		Benzylacetone	2550-26-7	0.20 ± 0.07	0.39 ± 0.14 *	35
54		6-Methyl-5-hepten-2-one	110-93-0	1.25 ± 0.70	nd	36
55		α-Citral	141-27-5	0.22 ± 0.06	nd	37
56		Humulene oxide II	19888-34-7	0.47 ± 0.20	nd	38

nd, no data; *, the *t*-test showed a significant difference in relative content compared to Zhutou galangal, *P* < 0.01. ^1^ Selected, the selected 38 compounds according to the two criteria described in Section 2.4.
